# Uncovering the roles of long non-coding RNAs in cancer stem cells

**DOI:** 10.1186/s13045-017-0428-9

**Published:** 2017-02-28

**Authors:** Xiaoxing Huang, Ruijing Xiao, Shan Pan, Xiangyong Yang, Wen Yuan, Zhenbo Tu, Ming Xu, Yufan Zhu, Qian Yin, Yingjie Wu, Weidong Hu, Liang Shao, Jie Xiong, Qiuping Zhang

**Affiliations:** 1 Department of Immunology, School of Basic Medical Science, Wuhan University, Wuhan, 430071 China; 2 Hubei University of Technology Engineering and Technology College, Wuhan, 430000 China; 3 Department of Oncology, Zhongnan Hospital of Wuhan University, Wuhan, 430071 China; 4 Department of Hematology, Zhongnan Hospital of Wuhan University, Wuhan, 430071 China

**Keywords:** Long non-coding RNAs, Cancer stem cells, Self-renewal, Malignant transformation, Tumor metastasis, Tumor recurrence

## Abstract

Cancer has been a major public health problem that has threatened human life worldwide throughout history. The main causes that contribute to the poor prognosis of cancer are metastasis and recurrence. Cancer stem cells are a group of tumor cells that possess self-renewal and differentiation ability, which is a vital cause of cancer metastasis and recurrence. Long non-coding RNAs refer to a class of RNAs that are longer than 200 nt and have no potential to code proteins, some of which can be specifically expressed in different tissues and different tumors. Long non-coding RNAs have great biological significance in the occurrence and progression of cancers. However, how long non-coding RNAs interact with cancer stem cells and then affect cancer metastasis and recurrence is not yet clear. Therefore, this review aims to summarize recent studies that focus on how long non-coding RNAs impact tumor occurrence and progression by affecting cancer stem cell self-renewal and differentiation in liver cancer, prostate cancer, breast cancer, and glioma.

**Electronic supplementary material:**

The online version of this article (doi:10.1186/s13045-017-0428-9) contains supplementary material, which is available to authorized users.

## Background

According to the result of Human Genome Project, only approximately 20 thousand genes of human genome have the encoding protein ability [1]. At the same time, only 5–7% of human genes can be stably transcribed. The vast majority of RNAs are therefore unable to be translated into protein and are termed non-coding RNAs [2–4]. Non-coding RNAs used to be considered by scientists to be “junk RNAs”; however, an increasing body of evidence suggests that non-coding RNAs play an important role in both physiological and pathological conditions [5–7].

**Fig. 1:**
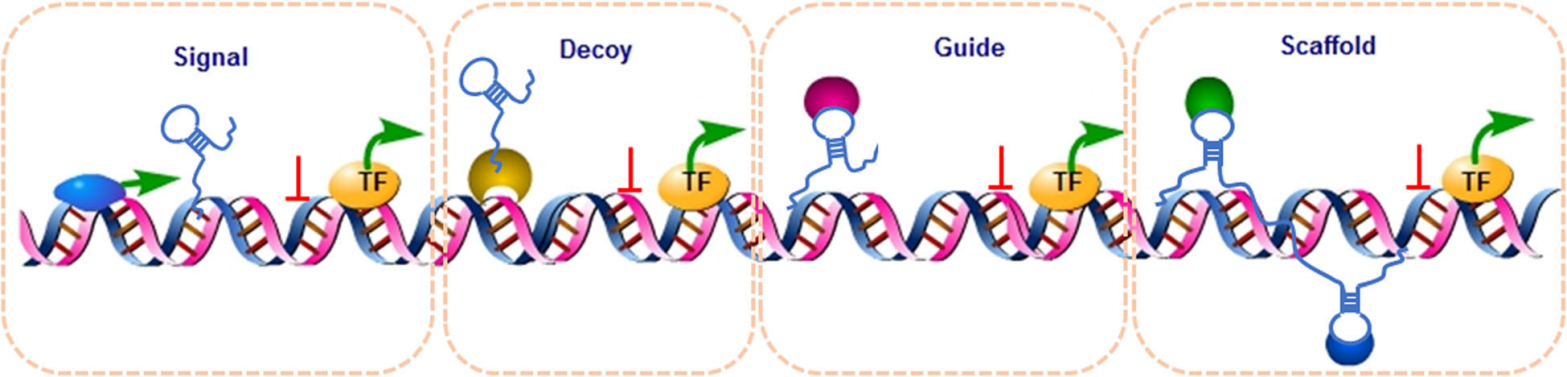
Four models of LncRNAs. Signal: LncRNAs can signal the space, time, and expression of gene transcription to modulate transcription factors and signaling pathways. LncRNAs directly bind to nucleic acid to inhibit the downstream molecule transcription named signal work model. Decoy: LncRNAs can bind and titrate away the protein or RNA target. LncRNAs combine with protein and then bind to nucleic acid to inhibit the downstream molecule transcription named decoy work model. Guide: LncRNAs can guide RNA-binding proteins to special target genes. Proteins guide LncRNA to bind to nucleic acid to inhibit the downstream molecule transcription named guide work model. Scaffold: LncRNAs can assemble different proteins to form complexes to initiate the special biological functions. Different proteins and LncRNAs combine together to bind to nucleic acid to inhibit the downstream molecule transcription named scaffold work model

Long non-coding RNAs (LncRNAs) are a class of non-coding RNAs that have no potential to code proteins and are more than 200 nucleotides in length. Portions of LncRNAs can be specifically expressed in different tissues and different cancers [8–10]. According to the literature, the disorder of LncRNAs is closely related to the occurrence and development of various cancers, such as leukemia, breast cancer, gastric cancer, colon cancer, liver cancer, lung cancer, and cholangiocarcinoma [11–17]. The GENCODE consortium (version 18) has confirmed the existence of 13,562 LncRNAs, and approximately 2/3 of them are located between genes, which are termed long intergenic ncRNAs (lincRNAs). Others include overlapping, antisense, and intronic LncRNAs [18]. Most LncRNAs are transcribed by RNA polymerase II to be spliced, polyadenylated, and 5′-capped [19, 20]. LncRNAs work mainly in four modes: signal, decoy, guide, and scaffold [21, 22]. They affect the transcription of genes and play a regulating role (Fig. 1) [22]. Signal: LncRNAs can signal the space, time, and expression of gene transcription to reflect the integrative biological outcome of transcription factors and signaling pathways controlling gene expression. Decoy: LncRNAs can bind and titrate away the protein or RNA target. Guide: LncRNAs can guide RNA-binding proteins to special target genes, either in the near or in the distant target genes. Scaffold: LncRNAs can assemble different proteins to form complexes to initiate the special biological functions [21, 22]. Compared with coding genes and other non-coding RNAs such as microRNAs (miRNAs), LncRNAs are highly conserved [23]. Their high conservation and versatility have made them important in cancer research in recent years.

The tumor complex contains heterogeneous cells of different differentiation degrees [24]. Cancer stem cells (CSCs) are a group of tumor cells that have differentiation and self-renewal ability [25]. In 2003, Visvader and Linderman first defined CSCs in acute myeloid leukemia [26]. Recently, more research has shown that CSCs are involved in the occurrence, development, invasion, metastasis, and drug resistance of numerous solid tumors [27–30], which is closely related to poor tumor prognosis. Removal of CSCs is considered a new hope for the eradication of malignant tumors and the prevention of tumor recurrence [31].

Some research has demonstrated that the aberrant expression of LncRNAs in malignant tumors is closely related to the function of CSCs. Numerous LncRNAs modulate the functions of CSCs by regulating OCT4, SOX2, KLF4, and other classic stem cell-related pathways [32–34]. In addition, other LncRNAs contribute to CSC functions and tumor occurrence and development by inhibiting the miR-200 family, let-7, miR-140, and other miRNAs [35–37]. In recent years, research regarding LncRNAs and CSCs gradually became important to cancer researchers. This review aims to summarize the current research status of LncRNA and CSCs in different tumors and to explore the significance of LncRNA in the removal of CSCs and tumor therapy.

### Hepatocellular carcinoma

Hepatocellular carcinoma (HCC) is a global health problem and is ranked sixth worldwide for malignant tumors [38]. Over 700,000 patients are newly diagnosed with HCC every year [39]. Thus, the mechanisms of HCC development and progression and therapeutic targets and methods have been the focus of scientists. Increasingly, research suggests that LncRNAs participate in the self-renewal and proliferation of HCC stem cells by different mechanisms and play important roles in HCC deterioration.

**Fig. 2:**
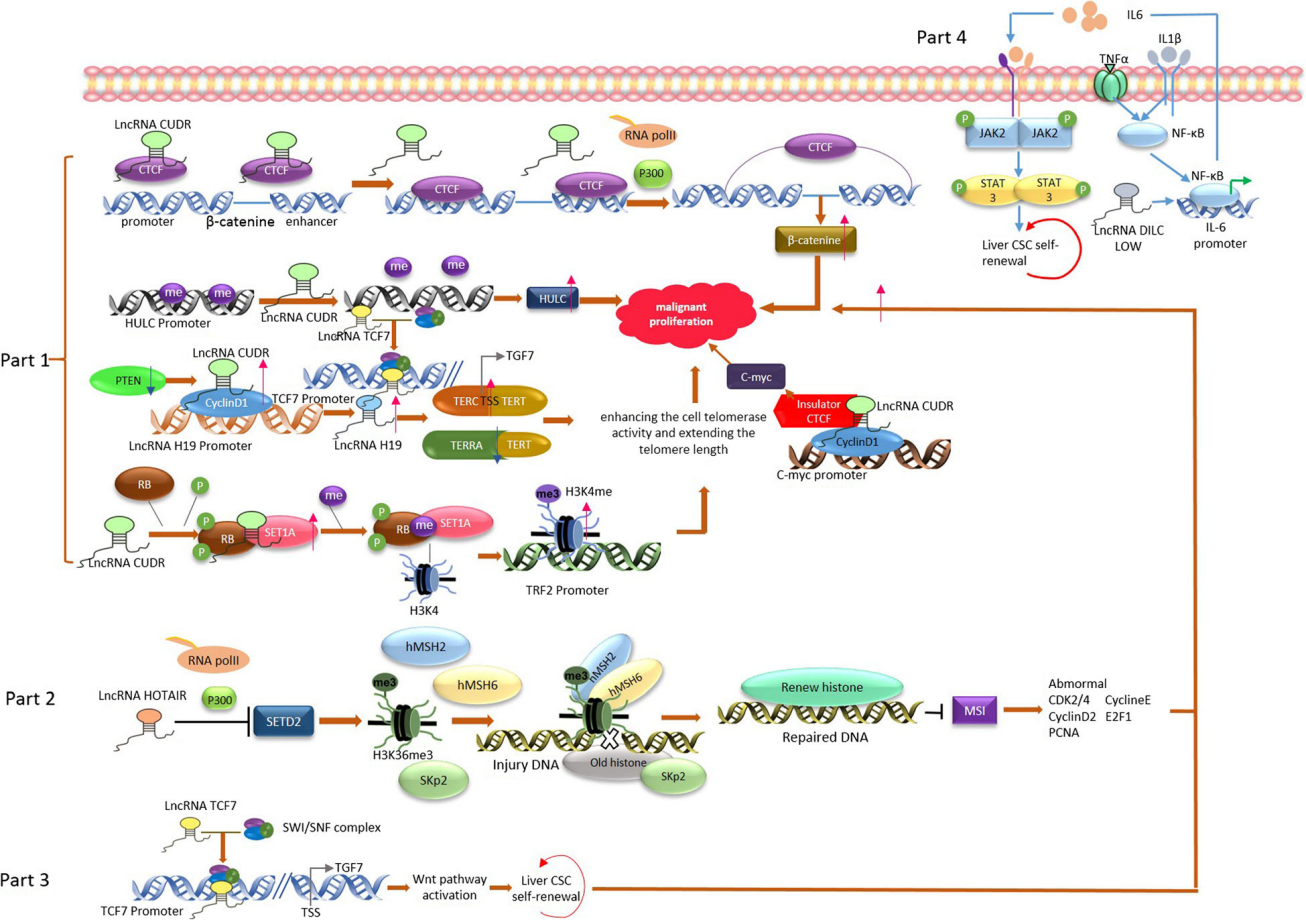
LncRNAs network in malignant transformation of HCC stem cells. *Part 1*: LncRNA CUDR impacts the malignant proliferation of liver CSCs trough different pathways. LncRNA CUDR act as oncogene by CUDR-HULC/CUDR-β-catenin signaling pathway; CUDR inhibits the methylation of gene promoter LncRNA H19 by combining with Cyclin-D to form the complex CUDR-CyclinD1, which upregulates the expression of LncRNA H19 and finally upregulates the expression of TERT and C-Myc to promote self-renewal and proliferation of HCC stem cells; LncRNA CUDR facilitate the interaction between SET1A and phosphorylated RB1 (pRB1) to be a complex contributing to the high level of H3K4 trimethylation, which took part in the malignant transformation of HCC stem cells via altering the length of telomere. *Part 2*: LncRNA HOTAIR can inhibit histone H3K36 trimethylation by suppressing SETD2. Then, to enhance the malignant proliferation of HCC stem cells via affecting the repair of aged histone, microsatellite stability, and cell cycle-related genes. *Part 3*: LncRNA TCF7 alter the expression of TCF7 by recruiting complex SWI/SNF to combine with TCF7 promoter region, which could activate WNT signaling pathways and accelerate self-renewal of HCC stem cells. *Part 4*: LncRNA DILC can inhibit liver CSCs’ self-renewal through the IL-6/STAT3 signaling pathway

According to previous research, the high trimethylation level of histone H3K4 and H3K27 was linked to low differentiation, a large volume of neoplastic foci, multiple tumors, vascular invasion, and poor prognosis of HCC [40, 41]. Li et al. found that LncRNA CUDR could enhance the interaction between SET1A and phosphorylated RB1 (pRB1) in HCC, and the complex they formed resulted in a high level of H3K4 trimethylation, which participated in the malignant transformation of HCC stem cells via altering telomere length [42]. At the same time, other research suggests that LncRNA CUDR can also function as an oncogene by the CUDR-HULC/CUDR-β-catenin signaling pathway [43]. Furthermore, CUDR inhibits the methylation of the gene promoter LncRNA H19 by combining with Cyclin-D to form the complex CUDR-CyclinD1, which upregulates the expression of LncRNA H19 and finally upregulates the expression of TERT and C-Myc to promote the self-renewal and proliferation of HCC stem cells [44] (Fig. 2 (Part 1)). Thus, it can be seen that LncRNA CUDR plays an important role in the self-renewal and proliferation of HCC stem cells by multiple signaling pathways.

In addition, Li et al. reported that LncRNA HOTAIR can inhibit the trimethylation level of its downstream histone H3K36 by suppressing the expression of SETD2, which can enhance the malignant proliferation of HCC stem cells and accelerate the progression of HCC by affecting the repair of aged histones, microsatellite stability, and cell cycle-related genes [45] (Fig. 2 (Part 2)). Thus, it can be seen that LncRNA can enhance the malignant transformation of HCC stem cells by regulating the abnormal modification of histones. At the same time, scientists have reported that LncRNAs can accelerate the development and progression of HCC by altering the classic signaling pathways that are related to HCC stem cells. For instance, lncTCF7 can alter the expression of TCF7 by recruiting SWI/SNF to combine with the TCF7 promoter region, which could activate the WNT signaling pathways and accelerate self-renewal of HCC stem cells and the deterioration of HCC [46] (Fig. 2 (Part 3)). LncRNA DILC can inhibit the self-renewal of HCC stem cells by blocking the HCC autocrine IL-6/STAT3 signaling pathway [47] (Fig. 2 (Part 4)). Moreover, LncRNAs can also take part in regulating the self-renewal and proliferation of HCC stem cells via inhibiting the combination of microRNA and target genes, playing a role in secretion in the tumor microenvironment [48, 49]. In conclusion, the results of several studies have indicated that LncRNAs play important roles in the malignant transformation of HCC stem cells, providing a new possibility for HCC clinical treatment.

### Prostate cancer

**Fig. 3:**
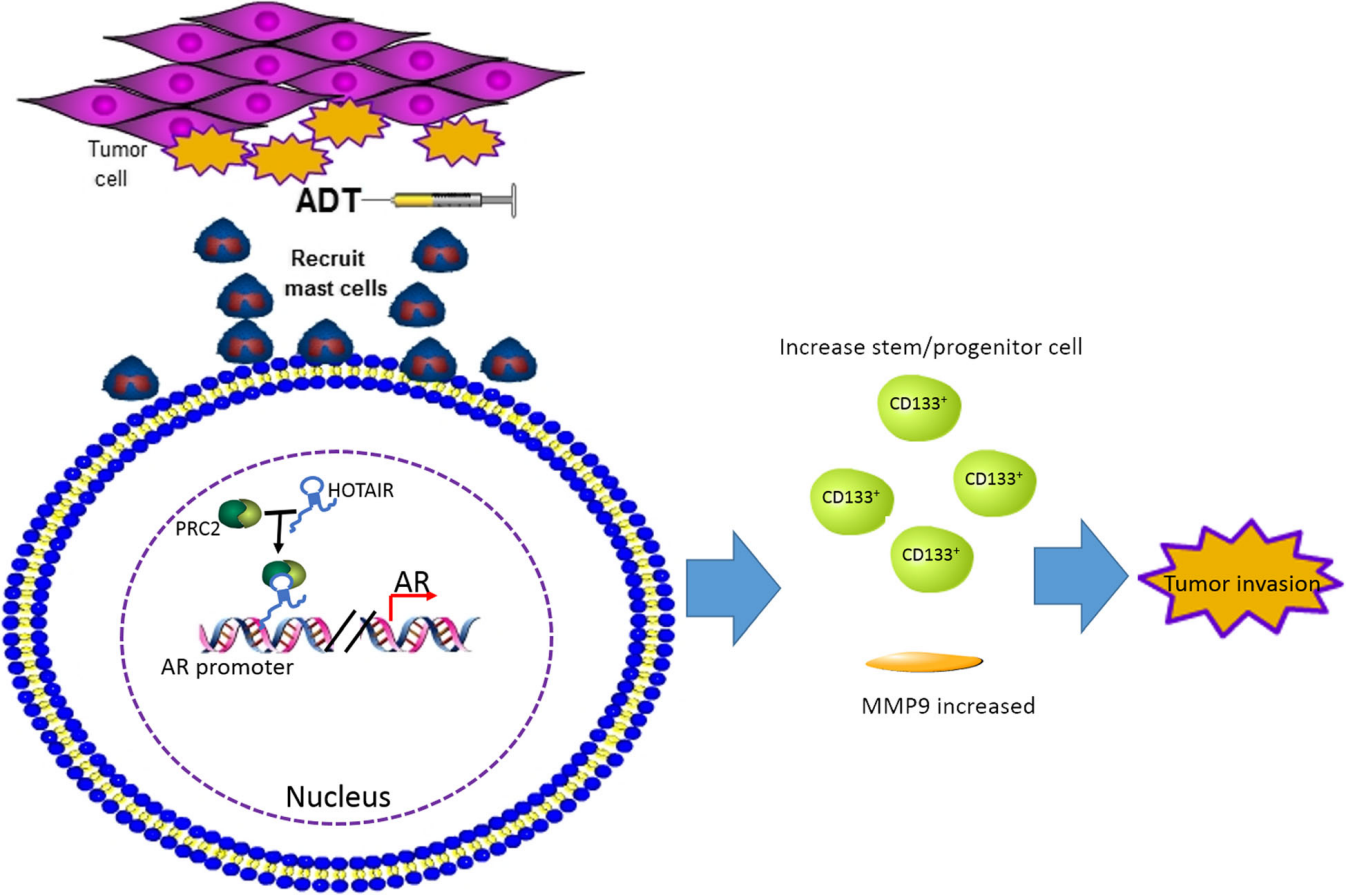
The roles of LncRNAs in malignant transformation of prostate CSCs. The PCR2-LncRNA HOTAIR complex which promotes by prostate tumor cells which treated with androgen-deprivation therapy (ADT) recruited mast cells to bind to androgen receptor (AR) gene to promote the invasion of prostate cancer through MMP9 and CD133^+^ stem/progenitor cells

Prostate cancer is the second most frequent cancer in the world [50]. Androgen receptor plays an important role in the occurrence and development of prostate cancer [51]. In the clinic, androgen blockade therapy is the standard treatment for prostate cancer, but androgen blocking resistance often appears after 1–2 years [52, 53]. Presently, research regarding the relationship between LncRNAs and prostate CSCs is still in the beginning stages. Scientists have found that LncRNA HOTAIR and LncRNA H19, which play important roles in liver CSCs self-renewal, can also affect the function of prostate CSCs. Lei Li and other researchers have found that after androgen blockade therapy in prostate cancer, a large number of mast cells can aggregate in the tumor microenvironment and then increase the number of stem cells and progenitor cells through the downstream androgen receptor signaling pathway. Mast cells enable the PCR2-LncRNA HOTAIR complex to bind to the 5′-flanking promoter region of the androgen receptor gene and thus suppress androgen receptor transcription, thereby affecting the expression of MMP9 and increasing CD133^+^ stem/progenitor cells to promote the invasion of prostate cancer [54] (Fig. 3). Forming complexes with extraneous molecules and then binding to the promoter of target gene to prevent its transcription is a common working model of LncRNAs. In prostate cancer, the inhibition of LncRNA H19 can repress the expression of classic stem cells related factors SOX2 and Oct4. At the same time, the inhibition of LncRNA H19 could repress the clone forming ability of prostate CSCs. This indicates that LncRNA H19 plays an important role in the maintenance of stemness in prostate CSCs [55]. Until now, studies regarding the functions of LncRNA on prostate CSCs are still limited, but it is believed that additional important LncRNAs will be found in prostate CSCs.

### Breast cancer

Breast cancer has long been a serious threat to the health of women around the world [56]. With in-depth studies on the occurrence and development of breast cancer, researchers have found that breast CSCs appear resistant to chemotherapy, radiotherapy, and hypoxia. Furthermore, breast CSCs exhibit high tumorigenicity and invasiveness, which are crucial to the occurrence, development, metastasis, and recurrence of breast cancer. According to the reports, LncRNAs can affect the characteristics of breast CSCs through various pathways, and have a great effect on breast cancer progression. During the occurrence and progression of breast cancer, the epithelial mesenchymal transition (EMT) is closely related to the function of breast CSCs, and numerous LncRNAs can promote or inhibit the metastasis and invasion of breast cancer cells by inducing or inhibiting EMT [57].

**Fig. 4:**
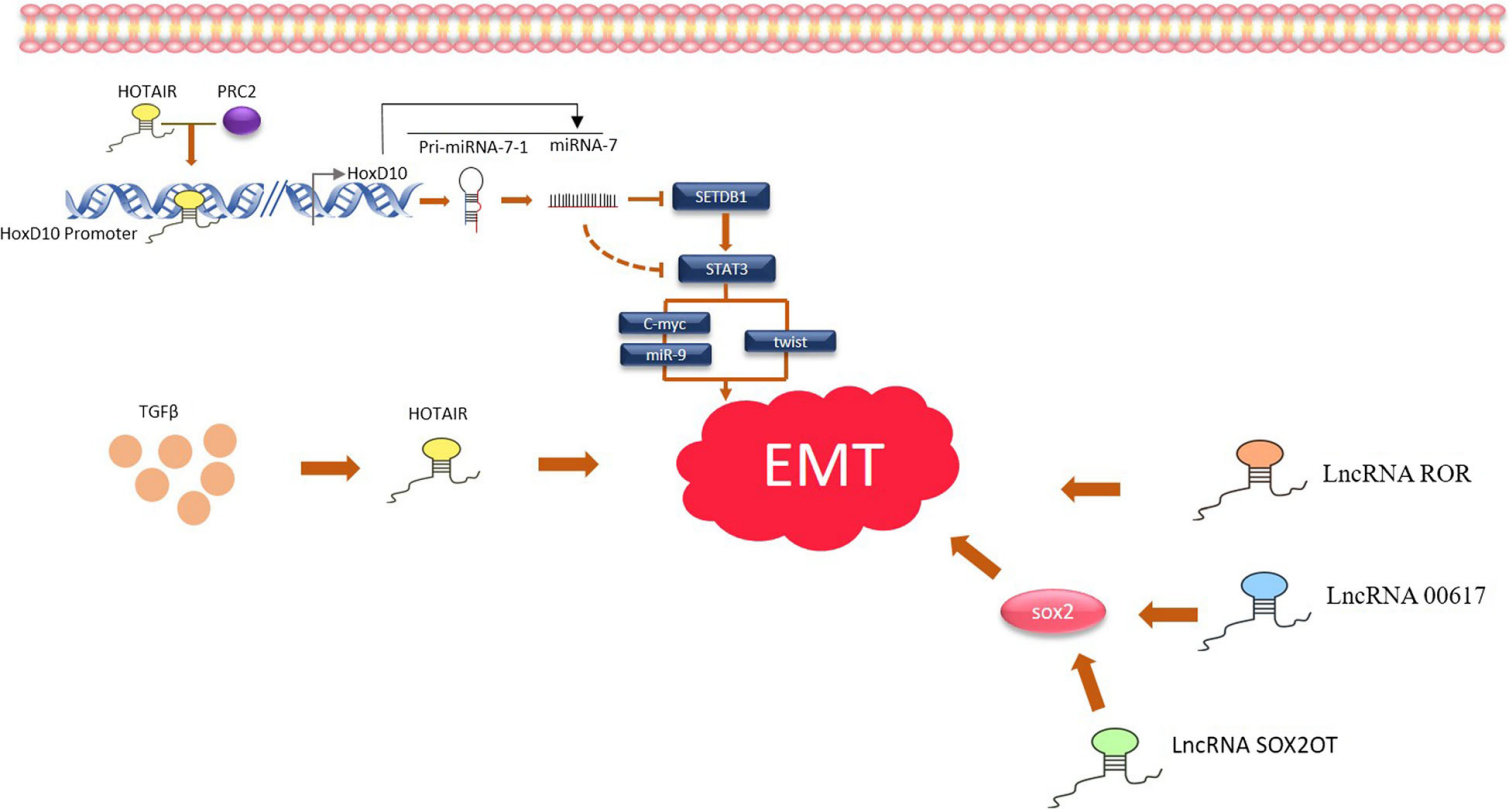
The roles of LncRNAs in the malignant transformation of breast CSCs. LncRNA HOTAIR affect the EMT phenomenon of breast cancer through HoxD10/miRNA-7/SETDB1/STAT3 pathway. At the same time, the secreted TGFβ could affect the expression of LncRNA HOTAIR. LncRNA 00617 and LncRNA SOX2OT contribute to EMT of breast cancer trough modulate SOX2. LncRNA ROR also could regulate the EMT of breast cancer

Pádua AC and other researchers reported that LncRNA HOTAIR played a key role in the occurrence of EMT in breast cancer cells. They found that treatment with the cytokine TGF-β1 can upregulate the expression of HOTAIR and induce the occurrence of EMT. While siRNA HOTAIR can inhibit the EMT induced by TGF-β1, it can also inhibit the cloning ability of breast cancer cells [58]. Moreover, Zhang et al. demonstrated that LncRNA HOTAIR can indirectly inhibit MIR-7, then depress SETDB1, and reverse the EMT of breast CSCs by downregulating the STAT3 pathway [59]. These studies indicate that LncRNA HOTAIR is a crucial factor in several EMT-related signaling pathways in breast cancer. Hou et al. found that LncRNA ROR is highly expressed in breast cancer tissues and is upregulated during EMT occurrence in human mammary epithelial cells. At the same time, the migration and invasion of breast cancer cells are enhanced and exert characteristics of primary stem cells. In contrast, silencing LncRNA ROR can inhibit the growth and lung metastasis of breast cancer cells [60]. H Li reported that LncRNA 00617 was highly expressed in breast cancer tissues and can promote the invasion and EMT in breast cancer cells by activating the transcription of SOX2. They showed that LncRNA 00617 exhibited carcinogenic activity in breast cancer [61]. SOX2 is crucial to the characteristics of CSCs [62]. Shahryari et al. showed that LncRNA SOX2OT is one of the main regulatory factors of SOX2 and found that aberrant expression of LncRNA SOX2OT and SOX2 appeared in numerous solid tumors such as breast and lung cancers [63]. This suggests that LncRNA SOX2OT may be related to the characteristics of breast CSCs (Fig. 4). Additionally, Zhou et al. reported that LncRNA-Hh can enhance the characteristics of CSCs in Twist-positive breast cancer through the Hedgehog signaling pathway [64].

In general, LncRNAs typically affect the characteristics of breast CSCs by influencing the EMT-related signaling pathways. These research achievements provide a broader space for us to study on breast CSCs. During the course of breast cancer treatment, LncRNAs could be used as new markers for early diagnosis and prognosis forecast in the future. In addition, they may also be a new therapeutic target and provide new ideas for breast cancer targeted therapy.

### Glioma

**Fig. 5:**
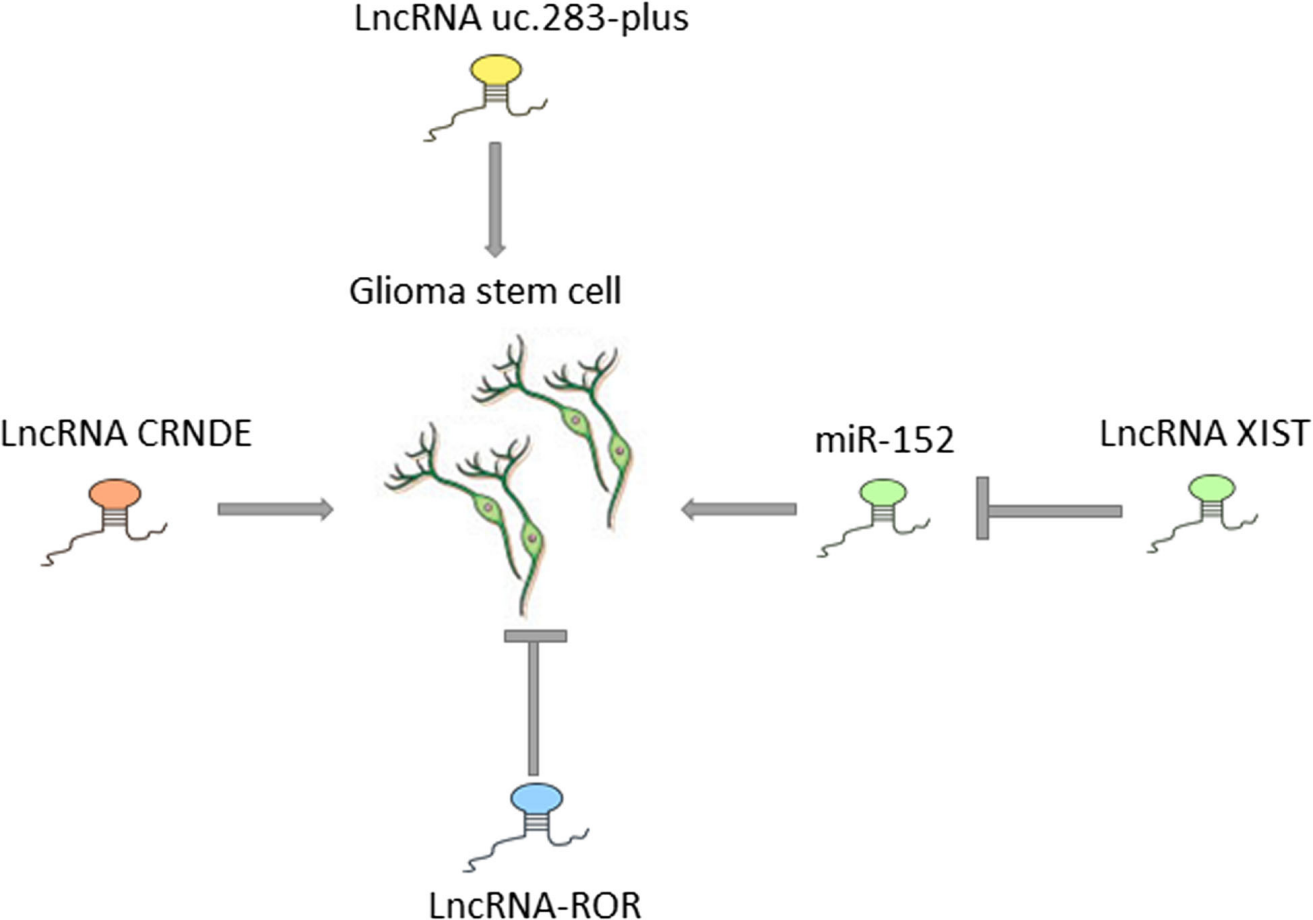
The roles of LncRNAs in the malignant transformation of glioma stem cells. LncRNA uc.283-plus, LncRNA CRNDE, LncRNA-ROR, and LncRNA XIST impact glioma stem cell functions through different pathways. Knockdown of the LncRNA XIST in glioma cells could lead to miR-152 upregulation, which resulted in the inhibition of CSCs

Glioma is the most common tumor in the central nervous system, and it exhibits a strong invasive ability. Glioma appears to diffusely infiltrate tissue, and the main current clinical treatment methods are surgery, radiotherapy, and chemotherapy, but their effect is limited. In recent years, studies have indicated that the expression of various LncRNAs are aberrant in glioma. Some of the LncRNAs showed increased expression in glioma compared with normal nervous tissues. Galasso et al. found that LncRNA uc.283-plus was specific to pluripotent stem cells, and it was highly expressed in glioma cells [65]. Ellis et al. found that LncRNA CRNDE with obvious stem cell-related properties is highly expressed in many kinds of tumor cells, including the glioma cells [66]. Yao et al. reported that LncRNA XIST was highly expressed in glioma stem cells, and the results of this study showed that knockdown of the LncRNA XIST in glioma cells could lead to miR-152 upregulation, which resulted in the inhibition of CSCs [67]. Nevertheless, some of the LncRNAs showed decreased expression in glioma. Feng S reported that the expression level of LncRNA-ROR in glioma tissues was significantly lower than the adjacent tissues. Additionally, LncRNA-ROR silencing could significantly enhance the proliferation of glioma cells and the spheronization of glioma stem cells. At the same time, the expression of LncRNA-ROR was negatively correlated with the expression of stem cell factor KLF4, which indicated that LncRNA-ROR played an inhibitory role in the proliferation and self-renewal of glioma stem cells [68] (Fig. 5). In summary, the aberrant expression of LncRNAs plays an important role in the malignant transformation and self-renewal of glioma stem cells. As research continues, LncRNAs may become new targets for the diagnosis and treatment of glioma.

## Conclusions

Research regarding the effect and mechanism of LncRNAs on CSCs’ self-renewal and malignant transformation is still in the initial stages. However, the existing research indicates that the aberrant expression of LncRNAs plays a key role in the self-renewal and malignant transformation of CSCs and tumor progression. At the present stage, research regarding the effect of LncRNAs on CSCs mainly concentrates on HCC, prostate cancer, breast cancer, and glioma, and researchers have found that the mechanisms of LncRNAs in different tumors are not consistent (see Additional file 1), which include changes in histone modification, the regulation of classic stem cell-related signaling pathways such as SOX2/KLF4, the induction of EMT, and the inhibition of miRNA function. These findings indicate that LncRNAs have strong biological effects on the function and mechanisms of CSCs. Additionally, numerous studies have found that LncRNA HOTAIR, LncRNA H19, and other classical LncRNAs could regulate the self-renewal and malignant transformation of CSCs through different mechanisms in different tumors. In general, the abnormal differentiation of CSCs is crucial to metastasis and recurrence in a variety of tumors, and LncRNAs play important roles in the abnormal differentiation of CSCs. However, up to now, the researches on cancer stem cells and LncRNAs have been focused on the influence of LncRNAs aberrant expression on cancer stem cells’ malignant transformation, but few studies have explored how LncRNAs change in the tumor environment. In the future, if we can clarify the relevant mechanisms, it will provide us with new ideas. We believe that the multiple functions of LncRNAs suggest that they may become novel promising targets to eradicate tumor stem cells, which may provide a new possibility for curative treatments of numerous cancers.

## Additional file

Additional file 1: Summary of LncRNAs related pathways in Hepatocellular carcinoma, Prostate cancer, Breast cancer, and Glioma. (XLSX 9 kb)

## Abbreviations

CSCs: Cancer stem cells
EMT: Epithelial mesenchymal transition
HCC: Hepatocellular carcinoma
lincRNAs: Long intergenic ncRNAs
LncRNAs: Long non-coding RNAs
